# Dynamics of Pathological and Virological Findings During Experimental Calpox Virus Infection of Common Marmosets (*Callithrix jacchus*)

**DOI:** 10.3390/v9120363

**Published:** 2017-11-28

**Authors:** Anne Schmitt, Li Lin Gan, Ahmed Abd El Wahed, Tingchuan Shi, Heinz Ellerbrok, Franz-Josef Kaup, Christiane Stahl-Hennig, Kerstin Mätz-Rensing

**Affiliations:** 1 Pathology Unit, German Primate Center (DPZ), Kellnerweg 4, 37077 Göttingen, Germany; Anne.Schmitt@bfr.bund.de (A.S.); fkaup@gwdg.de (F.-J.K.); 2 Unit of Infection Models, German Primate Center (DPZ), Kellnerweg 4, 37077 Göttingen, Germany; LGan@dpz.eu (L.L.G.); qinqin99413@googlemail.com (T.S.); stahlh@dpz.eu (C.S.-H.); 3 Division of Microbiology and Animal Hygiene, Department of Animal Sciences, University of Göttingen, Burckhardtweg 2, 37077 Göttingen, Germany; abdelwahed@gwdg.de; 4 Robert Koch-Institute, Centre for Biological Threats and Special Pathogens, Highly Pathogenic Viruses (ZBS1), Seestr. 10, 13353 Berlin, Germany; EllerbrokH@rki.de

**Keywords:** calpox virus, common marmoset, immunohistochemistry, nonhuman primate, orthopoxvirus, pathogenesis, transmission electron microscopy, virus infection

## Abstract

Experimental intranasal infection of marmosets (*Callithrix jacchus*) with calpox virus results in fatal disease. Route and dose used for viral inoculation of the test animals mimics the natural transmission of smallpox, thus representing a suitable model to study pathogenesis and to evaluate new vaccines against orthopoxvirus infection. However, the pathogenic mechanisms leading to death are still unclear. Therefore, our study aimed at investigating the kinetics of pathological alterations to clarify the pathogenesis in calpox virus infection. Following intranasal inoculation with two different viral doses, common marmosets were sacrificed on days 3, 5, 7, 10 and 12 post inoculation. Collected tissue was screened using histopathology, immunohistochemistry, transmission electron microscopy, and virological assays. Our data suggest that primary replication took place in nasal and bronchial epithelia followed by secondary replication in submandibular lymph nodes and spleen. Parallel to viremia at day 7, virus was detectable in many organs, mainly located in epithelial cells and macrophages, as well as in endothelial cells. Based on the onset of clinical signs, the histological and ultrastructural lesions and the immunohistochemical distribution pattern of the virus, the incubation period was defined to last 11 days, which resembles human smallpox. In conclusion, the data indicate that the calpox model is highly suitable for studying orthopoxvirus-induced disease.

## 1. Introduction

Although smallpox was successfully eradicated in 1980 [1], its causative agent, variola virus (VARV) still remains of concern because of the possible intentional release by bioterrorism [2,3]. Moreover, cessation of cross-protective smallpox vaccination has led to waning herd immunity, not only against VARV, but also against other orthopoxviruses (OPXV) [4]. As a consequence, human OPXV infections with monkeypox (MPXV) and cowpox (CPXV) are increasing [5,6,7].

MPXV is endemic in Central and Western Africa, with a 20-fold increase in human monkeypox incidence in the Democratic Republic of Congo over a 20-year period (between the 1980s and 2000s) [8,9]. In 2003, MPXV was accidentally imported into the US by MPXV-infected rodents from West Africa. The virus was transmitted via prairie dogs to some 40 humans [10]. In Europe CPXV infections in humans as well as in animals are also increasing [11,12,13,14]. Their ability to cross species barriers, to circulate among wild rodents and domestic animals, the limited options of therapeutics, and the severe course of disease in immunocompromised and atopic individuals, make CPXV a pathogen of public health concern [15,16,17,18]. In humans, the virus is often transmitted via skin or mucosal lesions from pet rats or cats, and causes self-limiting, painful hemorrhagic skin lesions, particularly located on the hands, face, or trunk in immune competent individuals [19,20]. CPXVs exist in many host species in Europe, but have also been reported in Israel and Egypt [21,22,23]. More than 30 outbreaks in zoological gardens have been reported over the last four decades [14,19,24,25,26]. Asymptomatic infected wild rodents seem to be the main natural reservoir for CPXV [27,28,29].

Therefore, development of medical interventions and new vaccine strategies and studies on pathogenesis are essential and require animal models, on which the efficacy of new vaccines and therapeutics can be tested. Over the last few decades, considerable progress has been made in developing small animal and nonhuman primate models with different OPXV, among them variola; but none fully mimic the natural route or course of infection in humans [30,31,32,33,34]. The advantage of small animal models is that larger numbers of animals can be used at lower maintenance costs compared to nonhuman primate models. However, the best species to evaluate new vaccines, therapeutics, or pathogenesis in an animal model, are nonhuman primates, due to their close relatedness to humans [35,36]. Owing to its high risk potential, research with VARV is restricted to two laboratories worldwide and there is an increasing international political interest in the eradication of the remaining VARV stocks. In July 2014, an unmarked box with smallpox virus was detected in a laboratory in Bethesda, Maryland, which has relaunched the debate about destroying all remaining virus stocks. The World Health Organization (WHO) has again postponed their decision for a destruction deadline, which is still under debate [37,38,39,40,41]. This uncertain future of VARV research requires development of other OPXV models [42]. CPXV-induced disease is an ideal model for OPXV, as experiments can be performed under Biosafety Level 2 laboratory conditions, in contrast to MPXV and VARV. Furthermore, CPXV shares homology with MPXV and VARV [43], and can induce disease in mice and NHPs [44,45].

In 2002, a lethal OPXV outbreak occurred in a German private husbandry [46], in which New World monkeys showed fever, lymphadenopathy, and severe erosive-ulcerative lesions of oral membranes. The isolated OPXV belonged to a small distinct CPXV cluster [19], and we designated it calpox according to its host *Callithrix jacchus*, the common marmoset [45]. Carroll and colleagues present in their study genotypic data of many different OPXV, among them the above described calpox virus (19). They conclude that the examined CPXV belong to five groups: groups 1 to 4 describe CPXV-like clades and one further group describes VACV-like clades. Calpox virus is grouped together with CPXV, which was isolated in 1980 from a local skin lesion of an elephant in Germany [47]. By experimental infection of common marmosets with calpox, a new animal model for OPXV disease was established. Compared to other animal models, intranasal application of very low doses of 5 × 10^2^ plaque-forming units (PFU) led to a severe systemic disease comparable to natural OPXV infection [45].

Previous studies in which the pathology of calpox was investigated, focused on the final stage of disease, in which characteristic pox-like lesions were detected in skin, mucous membranes, lymph nodes, liver and spleen [48]. Here we describe a pathogenesis study starting with analyses early after inoculation and looking for primary replication sites, cell tropism, and the progress of systemic viral spread at defined time points following infection. Our findings suggest that upon intranasal inoculation, the viral portals of entry are the nasal mucosa and bronchi.

## 2. Material and Methods

### 2.1. Experimental Animals

Overall, 21 healthy, sexually mature common marmosets (*Callithrix jacchus*) of different age and either sex from the breeding colony of the German Primate Center were used in this study (see Table 1). They were kept under Biosafety Level 2 conditions, received a complete balanced diet and water ad libitum, and were provided with standard environmental enrichment. All experimental procedures complied with the German Animal Welfare Act. The study was approved by the responsible veterinary authorities (approval number 33.9-42502-04-12/0745, 19 April 2012) and was conducted in accordance with European Union (EU) guidelines for the accommodation and care of animals used for experimental and other scientific purposes. During the study, animals were assessed by experienced animal care takers twice a day for any signs of distress, pain, or sickness, by inspecting water and feed intake, feces consistency, and general condition. Measurement of the body temperature was not possible. In case of any unusual presentation, animals were attended by veterinarians.

### 2.2. Virus, Infection and Necropsy

The calpox virus used in this study originated from an outbreak in a private husbandry in 2002 [46]. It was propagated in Hep2 cells and stored in aliquots at −80 °C until use. Plaque assays were carried out to determine the median tissue culture infectious dose of the virus stock before in vivo use [45]. For virus inoculation, animals were anaesthetized by intramuscular injection of 0.1 mL of a mixture of 5% ketamine, 1% xylazine and 0.01% atropine per 200 g body weight. Animals euthanized on day 5, 7, 10, and 12 were infected intranasally [45] with 8.3 × 10^3^ PFU of calpox virus. This dose was chosen because it reliably led to infection in 6 out of 6 control animals from unrelated studies [45]. To increase the likelihood for the identification of the port of viral entry, the animals euthanized on day 3 were inoculated an approximately 40-fold higher dose (3.5 × 10^5^ PFU) by the same route. Animals were monitored daily for the appearance of disease symptoms. At defined time points (see Table 1) animals were humanely euthanized by premedication with the anesthesia mixture followed by an intraperitoneal overdose of pentobarbital (Narcoren, Fa. Merial GmbH, Hallbergmoss, Germany). To compare the histological alterations observed during the incubation period with those after onset of disease, a further six animals (serving as untreated controls in a vaccine study) were included. They were euthanized due to animal welfare reasons, as they met endpoint criteria or died spontaneously.

### 2.3. Microscopy

Full necropsy was performed to collect samples from 30 organs. Samples were fixed in 4% neutral buffered formalin, processed routinely and embedded in paraffin. Sections were stained with haematoxylin and eosin using the Varistain Gemini automated stainer (Thermo Fisher Scientific, Frankfurt/Main, Germany). To determine the distribution of calpox virus, immunohistochemistry (IHC) was performed using a streptavidin alkaline phosphatase detection kit (Roche, Mannheim, Germany) on 2–4 µm thick sections of formalin-fixed paraffin embedded tissue. Sections were loaded into a DiscoveryXT autostainer (Roche, Mannheim, Germany), deparaffinized, rehydrated, and incubated with a rabbit anti-vaccinia virus polyclonal antibody (purified IgG fraction, 1:2000 dilution; Catalog #YVS8101, Accurate Chemical, Westbury, NY, USA) for 32 min at 37 °C followed by incubation with the secondary antibody (1:500 dilution, biotinylated immunoglobulin (Ig) cocktail of goat anti-mouse IgG, goat anti-mouse IgM, goat anti-rabbit IgG and protein block) for 20 min. Fast Red (Roche, Mannheim, Germany) was used as the substrate chromogen and slides were counterstained with hematoxylin before examination by light microscopy. Tissue sections from previously confirmed calpox virus infected marmosets were used as positive control samples. Negative control staining was performed by omission of the primary antibody on the infected tissue and by using tissue of uninfected animals. Additionally, selected tissues were fixed in 2.5% glutaraldehyde, embedded in epon, and prepared for transmission electron microscopy (TEM).

### 2.4. Histologic and Immunohistochemistry Scoring

Histological lesions were graded semiquantitatively according to the following scale: Grade 1 represented very mild lesions; Grade 2 represented lesions which were more severe in their character but did not affect the whole organ; Grade 3 represented severe lesions that involved most parts of the organ.

We employed a semiquantitative scoring method that assigned immunohistochemistry scores as a percentage of calpox virus positive cells with slides being scored as negative, very weakly positive (+, only scattered cells), mildly positive (+, staining in less than 5% of cells per high power field), moderately positive (++, staining in less than 50% of cells), and strongly positive (+++, staining in more than 50% of cells). Negative control slides (without primary antibody and tissue of non-infected animals with primary antibody) did not show any specific reaction.

### 2.5. Microbiological Examinations

Immediately after opening the body cavities, bacteriological smears were collected from different organs (heart, lung, liver, spleen, kidney, brain, small and large intestine) with a sterile inoculation loop, spread on nutrient agar (blood agar, Columbia agar, MacConkey agar, SS-agar, Sabouraud agar) and incubated at 37 °C for 24 h. Isolated bacteria were characterized biochemically. The E. coli strains were typed as serovar O2:H14 by a reference laboratory.

### 2.6. Isolation of Calpox Virus DNA from Tissues

After weighing each tissue sample, phosphate buffered saline (PBS) was added at a ratio of 1:10 as well as a 5 mm diameter stainless bead. The TissueLyser II (Qiagen, Hilden, Germany) was then used for homogenization. The homogenization times varied among tissues ranging from 20 s (brain and bone marrow) to three rounds of 1 min (skin). After centrifugation of the homogenates at 125 g for 10 min, the supernatant was removed. 100 µL-aliquots each were used for DNA extraction and an endpoint dilution assay. In few cases, not all organs were available for homogenization (see Figure 3). For DNA extraction from tissues, the QIAamp^®^ DNA Mini Kit (Qiagen, Hilden, Germany) was used. DNA was eluted in 100 µL AE buffer (Qiagen, Hilden, Germany) and stored at −20 °C.

### 2.7. Isolation of Calpox Virus DNA from Blood

For extraction of Calpox virus DNA from blood, 100 µL of whole blood containing ethylenediaminetetraacetic acid were diluted 1:2 with PBS. Extraction followed the manufacturer´s protocol of the QIAamp^®^ DNA Mini Kit (Qiagen). Elution and storage was the same as for tissue DNA preparation.

### 2.8. Analysis of Viral Load by Real-Time PCR

In order to determine the load of calpox virus in various organs and body fluids, a real-time PCR targeting the gene region of the ankyrin repeat-containing protein (GenBank accession number HQ420898) was used. A quantified molecular DNA standard with the target DNA cloned into a TOPO-TA vector (Invitrogen, Karlsruhe, Germany) was applied to calculate the concentration of calpox viral DNA either per gram of tissue or per microliter of blood. The TaqMan^®^ Universal PCR Master Mix (Thermo Fisher Scientific (formerly Life Technologies), Darmstadt, Germany) was used as follows: 7.5 µL of TaqMan^®^ Universal PCR Master Mix was mixed with 1 µL of each primer at a concentration of 10 nmol (Calp1for: 5′-CCggCATgCgTgACTgAATT-3′ and Calp1rev 5′-TAAgATgCgAgCCgAgAAgC-3′), 0.5 µL of the 10 nmol of TaqMan probe TIB-1 (FAM -TgCTCCgTgTTCTACCATCgTgCg-TAMRA), 4 µL molecular biology grade water and 1 µL of the extracted DNA. Oligonucleotides were synthesized by TIB Molbiol (Berlin, Germany). The real-time PCR was performed using the 5-Plex Rotor-Gene Q (Qiagen, Hilden, Germany) and the following cycling conditions were applied: initial activation at 95 °C for 10 min, and 45 cycles of 94 °C for 15 s, and 58 °C for 45 s. Absolute quantification was conducted via Rotor-Gene Q Series Software 2.1.0 (Qiagen, Hilden, Germany).

### 2.9. Quantification of Infectious Virus by Endpoint Dilution Assay

Infectious calpox virus in tissue homogenates was determined by endpoint dilution assay. For that purpose, 100 µL of the tissue homogenate (see: Isolation of calpox virus DNA from tissues) was used. The tissue homogenates were ten-fold serially diluted with Dulbecco’s modified Eagle’s medium (DMEM), 4.5 g/L glucose (PAN-Biotech) with 2% fetal calf serum (FCS) and a mixture of antibiotics (1% penicillin-streptomycin; 50 µg/mL neomycin; 100 µL/mL nystatin; 1 µg/mL amphotericin and 1% gentamycin). 100 µL of each dilution were added to Vero E6 cells pre-seeded in quadruplicate in 96-well (MT) plates and allowed to incubate at 37 °C and 5% CO_2_ for four days. Thereafter, viral antigen was visualized by intracellular immunoperoxidase staining. Briefly, cells were permeabilized with methanol for 15 min. at −20 °C. Then plates were washed with PBS three times, and unspecific protein binding sites blocked with PBS containing 2% milk powder for 1 h at 37 °C. For all further dilutions, PBS containing 2% milk powder was used. Next, 50 µL of optimally diluted human hyperimmune serum against VACV (kindly provided by Claus-Peter Czerny, University of Göttingen, Göttingen, Germany) was added to each well. After 1 h incubation at 37 °C plates were washed three times with PBS and 50 µL of a 1:1000 dilution of goat anti-human IgG peroxidase conjugate (Jackson ImmunoResearch, West Grove, PA, USA) was added to each well. Following 1 h incubation at 37 °C plates were extensively washed with PBS and a chromogen-substrate mixture was added (for one MT plate 2 mg 3-amino-9-ethylcarbazole dissolved in 300 µL dimethylformamide added to 5 mL 50 mmol sodium acetate and 25 µL 3% H_2_O_2_). Color development was stopped after 20 min by washing with distilled water and the cells were scored under a light microscope, with infected cells being identified by a dark brown cytoplasmic staining. Results are given as 50% tissue culture infective dose (TCID_50_)/g tissue.

## 3. Results

### 3.1. Clinical Findings

Animals which were euthanized at predetermined time points within the first 10 days did not show any clinical signs of disease. However, the animals which were euthanized or died spontaneously between 12 and 16 days after inoculation (dpi), showed varying symptoms. Two out of three animals from the group of predefined euthanasia on day 12 had only very few typical pox lesions on skin and mucocutaneous junctions and mild nasal discharge while the third one (animal no. 15) presented with severe clinical disorders, i.e., fatigue and hemorrhagic nasal discharge on the day before necropsy and additionally hemorrhagic urine, hemorrhagic anal discharge and multiple pox lesions on skin and mucocutaneous junctions on the day of necropsy.

### 3.2. Gross Necropsy Findings

Comprehensive post mortem examinations were performed on days 3, 5, 7, 10 and 12 (*n* = 3/time point), or when the animals died spontaneously or met the endpoint criteria on day 12 (animal no. 16, 17), 13 (animal no. 18), 14 (animal no. 19, 20) and 16 (animal no. 21). Within the first 10 days, only very discrete lesions concerning the lymphoid system were observed. Mild lymphoid hyperplasia of the spleen, the submandibular and axillary lymph nodes were the most prominent lesion. Final stage animals revealed seromucous nasal discharge, focal erosive-ulcerative gingivitis coinciding with loss of incisors (animal no. 18, 19, 21), and typical calpox lesions on skin, mucocutaneous junctions and larynx (animal no. 17), consisting of erythema or vesicular lesions distributed over the whole body (lips, eyelid, thighs, arm, abdomen, thorax, inguinal and anogenital region). Subcutaneous tissue of one animal was moderately edematous (animal no. 17). Additional findings included multifocal hemorrhages in the lung (animal no. 15), moderate splenomegaly, severe lymphoid hyperplasia and hyperemia of submandibular, axillary, and inguinal lymph nodes, hemorrhages in the mesovarium (animal no. 17), scrotum (animal no. 13, 15), tonsil (animal no. 21), and the hamstring muscle (animal no. 21), focal hyperemia of small and big intestine (animal no. 13, 15), hemorrhagic cystitis (animal no. 13, 15, 16, 21), and mild serosanguinous pericardial effusion (animal no. 13, 15).

### 3.3. Histopathology and Immunohistochemistry

Tissues were examined by light microscopy and histopathological and immunohistochemical findings in selected organs are summarized in Table 2 and Table 3.

**Figure 1:**
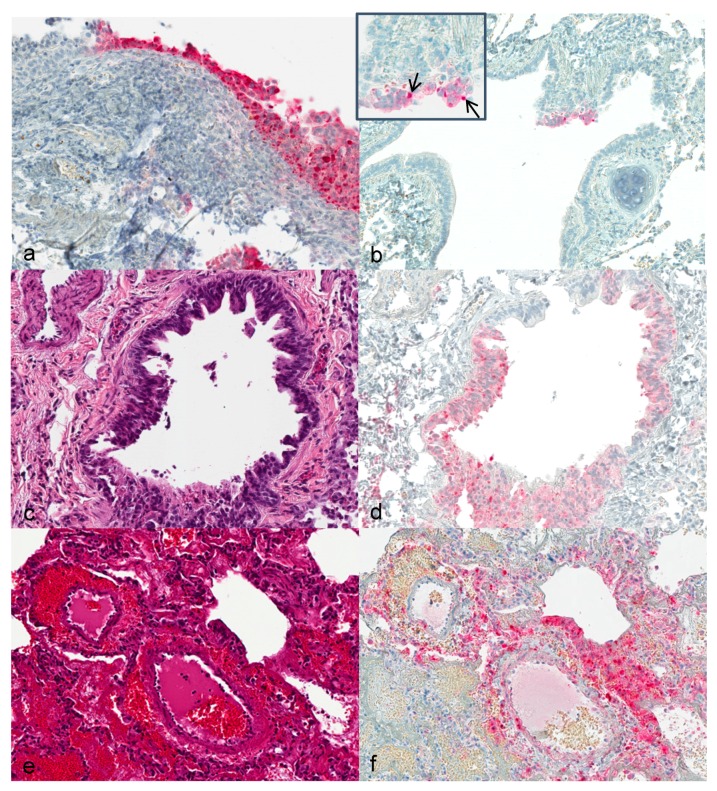
Histological and immunohistological findings in the respiratory tract. (**a**) Animal no. 3. Nasal epithelium, common marmoset, 3 dpi with moderate hyperplasia and necrosis of epithelial cells and strong signal for viral antigen in epithelial cells. Immunohistochemistry (IHC); (**b**) animal no. 2. Lung, common marmoset (*Callithrix jacchus*), 3 dpi with focal immunohistochemical detection of viral antigen in bronchial epithelium. Inset. Higher magnification of respiratory epithelium with clearly visible intracytoplasmic inclusion bodies (arrows). IHC; (**c**) animal no. 9. Lung, common marmoset, 7 dpi. Bronchus with severe hyperplasia, necrosis, and desquamation of respiratory epithelium. Hematoxylin and eosin (HE); (**d**) same animal, same location as c. Intracytoplasmic inclusion bodies in respiratory epithelium are strongly immunopositive. IHC; (**e**) animal no. 15. Lung, common marmoset, 12 dpi. Diffuse perivascular and intraalveolar hemorrhage with activation of endothelial cells. HE; (**f**) same animal, same location as f. Strong immunoreactivity in endothelial cells and in alveolar histiocytes. IHC. Magnification: 40×.

Upper respiratory tract: First alterations in the nasal ciliated epithelium were visible 3 dpi. Severity of alterations increased with duration of infection. At 12 dpi, extensive necrosis and acanthosis of ciliated epithelium with intracytoplasmic inclusion bodies were seen. Immunohistochemically, calpox specific antigens were first seen in respiratory epithelium (3 dpi) and additionally in endothelial cells, collagen fibers and histiocytes at later time points (Figure 1a).

Lung: Hyperplastic bronchiolar epithelial cells were observed at 3 dpi with detection of viral antigen (Figure 1b). By day 7, epithelium presented with acantholysis and showed signs of acute necrosis (Figure 1c). At 12 and 14 dpi multifocal bronchopneumonia with degeneration and acute necrosis of bronchiolar epithelium (Figure 1e) was observed. Viral antigen was detected in bronchiolar epithelial cells at 3 dpi in the lung of animals that were infected with the higher dose. From day 7 to 12 onwards, viral antigen was seen in the lungs of all animals (Figure 1d,f).

**Figure 2:**
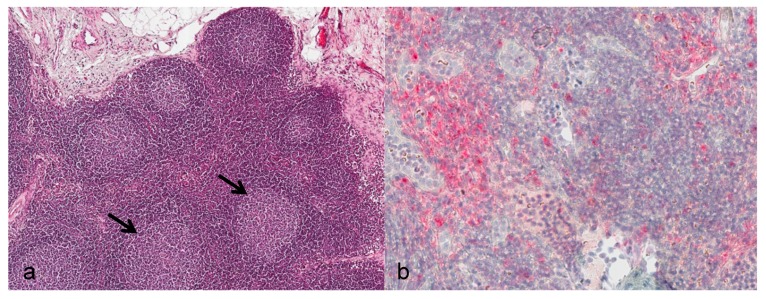
Histological and immunohistological findings in the lymphatic system. (**a**) Animal no. 4. Submandibular lymph node, common marmoset, 5 dpi. reactive lymphoid hyperplasia, follicular pattern, with enlarged follicles and reactive germinal centers (arrows). Magnification: 20×. HE; (**b**) animal no. 12. Submandibular lymph node, common marmoset, 10 dpi. Multifocally distributed immunopositive cells. IHC. Magnification: 40×.

Lymph nodes: During the asymptomatic phase, the enlarged submandibular lymph nodes showed reactive lymphoid hyperplasia of the follicles (Figure 2a). From day 12 onwards, severe alterations consisting of necrotizing lymphadenitis with diffuse hemorrhage, lymphoid depletion, capsulitis, and partly basophilic bacterial colonies were evident. Positive immunostaining for viral antigen was seen in cortical regions directly subjacent to the subcapsular sinus starting at day 7. By day 10, diffuse immunostaining was noted (Figure 2b).

Spleen: Splenic changes resembled those seen in submandibular lymph nodes: Mild to marked follicular hyperplasia within 10 dpi, and by 12 dpi severe necrotizing alterations with lymphoid depletion and partly with basophilic bacterial colonies were observed. Calpox viral antigen was detected in one animal by 7 dpi, and at later time points in periarteriolar lymphoid sheaths, endothelial cells, and mesothelial cells of the splenic capsule.

Liver: Only mild histopathological alterations (hepatocellular vacuolation) were observed within 10 dpi. However, multifocal coagulation necrosis and degenerated hepatocytes with eosinophilic intracytoplasmic inclusion bodies were obvious by day 12. Calpox viral antigen was detectable in scattered Kupffer cells within the sinusoid, beginning on day 10. By day 12, numerous hepatocytes showed calpox specific immunoreactivity and inclusion bodies. However, two animals in the later stage did not reveal any labelled cells in the liver at all.

Skin: Skin lesions typical for calpox virus were not detectable before 12 dpi. The prominent lesions consisted of intraepidermal vesicles with serocellular crusting, and ballooning degeneration of adjacent keratinocytes. Many keratinocytes of all cell layers contained eosinophilic intracytoplasmic inclusion bodies. The inflammatory cell infiltrate consisted of mainly mononuclear and few neutrophilic cells. The inclusion bodies consistently showed a strong immunoreactivity for calpoxviral antigen.

Further lesions which were observed at the late stage of disease comprised hemorrhagic cystitis (*n* = 3) with viral antigen present in transitional epithelium, macrophages and fibroblasts; hemorrhages within subcutaneous tissue of the scrotum (*n* = 2) and within the mesovarian tissue (*n* = 1); hemorrhage of the hamstring muscle (*n* = 1) with proof of viral antigen in spindle-shaped cells; and inflammatory cell infiltrates within the zona fasciculata of the adrenal gland (*n* = 3) with immunopositive inclusion bodies within epithelial cells.

To sum up, only discrete virus-associated lesions were observed within the first 10 days after infection. The primary target organ with proof of intralesional viral antigen was the nasal epithelium (3 dpi). Submandibular lymph nodes showed follicular hyperplasia along with the presence of viral antigen at day 7. At 10 dpi, viral antigen was detectable within the liver, i.e., Kupffer cells. From day 12 onwards, virus-associated pneumonia, hepatitis, dermatitis, splenitis, and lymphadenitis with lymphocytic depletion were seen (see Table 2).

### 3.4. Transmission Electron Microscopy

Ultrastructurally, virus was not detectable before day 12. Transmission electron microscopy revealed developing and mature stages of calpox virus in mononuclear and epithelial cells of lung, skin, buccal mucosa, liver, adrenal gland, uterus, spleen, bone marrow, and lymph nodes. Virus factories (B-type inclusions) with virion assembly and maturation, as well as mature viral particles in the cytoplasm and in A-type inclusions (ranging from 0.5 µm to 9 µm in diameter) were noticed. The appearance of the immature stages varied from crescent structures to circular forms containing electron-dense, granular material. At later stages the nucleoprotein condensated and a dense dumbbell-shaped core arose. Mature enveloped viral particles averaging 300 nm in length were oval to brick shaped. The core was flanked by lateral bodies.

### 3.5. Microbiological Examination

Four animals were found to be positive for bacteria in several organs by culturing tissue smears on blood agar at necropsy. Two animals revealed growth of hemolyzing *Escherichia coli* (*E. coli*) in heart, lung, spleen, liver, brain and kidney. One animal with severe growth of *E. coli* showed additional growth of *Klebsiella pneumoniae* in heart and lung (animal no. 16). The other animal showed only mild growth of *E. coli* (animal no. 21). From the spleen of one monkey, a few colonies of hemolyzing *E. coli* were isolated (animal no. 18) and another monkey showed germs in the heart, lung, spleen, liver, brain, and kidney (animal no. 17). To further identify the *E. coli* strain, single colonies were analyzed and typed as serovar O2:H14.

### 3.6. Calpox Virus Load in Different Tissues

**Figure 3:**
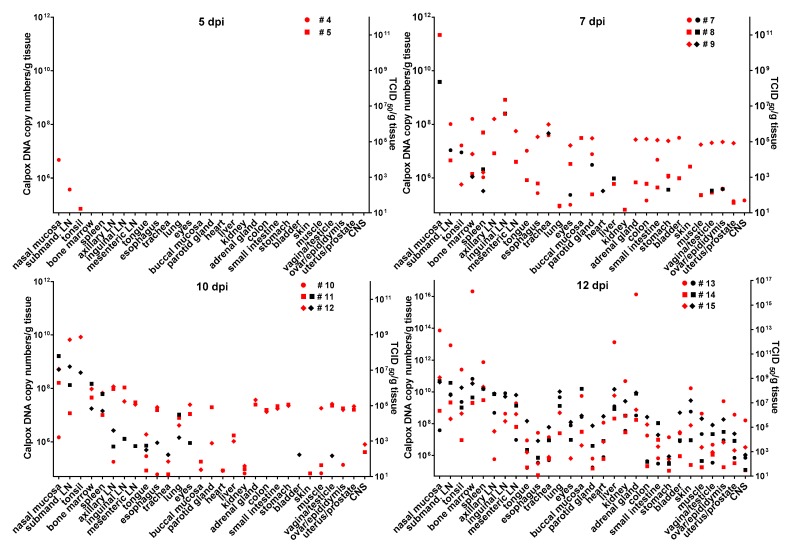
Calpox viral load in organs during the presymptomatic phase following intranasal inoculation with a low dose of calpox virus (8.3 × 10^3^ PFU). Viral load was determined by real-time PCR and endpoint dilution assay at the indicated time points of necropsy and expressed as calpox DNA copy numbers (red symbols) and infectious calpox virus titer (black symbols) per gram tissue, respectively. Systemic viral spread was observed by day 7 post inoculation. Highest viral loads were seen at day 12. In one animal sacrificed at day-5, no calpox copy numbers were detectable and all day-5 animals were negative for infectious virus. The following organs were not available for analysis: tonsil in animal no. 8, inguinal lymph node in animal no. 14, and skin in animal no. 4, 6 and 12. Animal numbers are indicated. CNS is central nervous system; dpi, days post infection; LN, lymph node; submand., submandibular; TCID_50_, 50% tissue culture infectious dose. Of note is the different scaling of the y-axes of the lower right panel due to the detection of higher viral loads.

**Figure 4:**
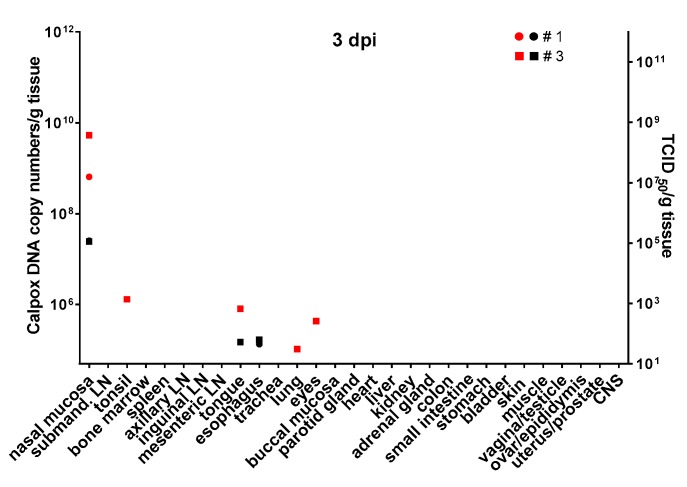
Calpox viral load in various organs three days following intranasal inoculation with a high dose of calpox virus (3.5 × 10^5^ PFU). Viral load was determined by real-time PCR and endpoint dilution assay and expressed as calpox DNA copy numbers (red symbols) and infectious calpox virus titer (black symbols) per gram tissue. In two of the three animals, viral DNA and infectious virus were detectable at the inoculation site, i.e., nasal mucosa, or in organs mostly close to it. Highest viral loads were measured in the nasal mucosa. Animal no. 2 remained negative for viral copy numbers and infectious virus. For abbreviations see legend of Figure 3.

Infectious calpox virus in tissues was quantified by endpoint dilution assay and compared to real-time PCR results using the same tissue preparation. Data obtained by the two different techniques were normalized to one gram of tissue. It was difficult to predict at which time point after inoculation we would be able to find first traces of virus with our assays. Thus, we started sacrificing animals five days after inoculation with 8.3 × 10^3^ PFU of calpox virus. Indeed, low levels of viral DNA (7 × 10^4^–4.7 × 10^6^ copy numbers/gram tissue) were detectable in two out of three animals, i.e., in one in the tonsil, and in the other in nasal mucosa and submandibular lymph node (Figure 3, upper left panel), but no virus was isolated from any organ. Next, we euthanized animals inoculated with the same dose at days, 7, 10 and 12. In animals sacrificed one week after inoculation, our findings indicated a systemic spread of the virus. At this time, when considering the findings from the three animals collectively, all organs tested were found to be positive for viral DNA, from half of them infectious virus was isolated. Highest viral loads were observed in the nasal mucosa of one animal (animal no. 8: 2.2 × 10^11^ DNA copies and 2.3 × 10^8^ TCID_50_). In the other organs, viral loads were lower ranging from around 10^5^–10^9^ DNA copy numbers and 10^2^–4 × 10^7^ TCID50 (Figure 3, upper right panel). By day 10, viral loads did not seem to have increased compared to those observed in the day-7 animals (Figure 3, lower left panel). On this day of necropsy, infectious virus was detectable in only around 50% of the tissues, and levels of infectivity were the same (animal 11, nasal mucosa), or between one and five orders of magnitude lower compared to DNA copy numbers. Overall, highest viral loads were detected in the day-12 animals, reaching up to 10^16^ copy numbers (animal 13, nasal mucosa, bone marrow and adrenal gland) and up to 10^9^ infectious units (animal 13, bone marrow). It is of note that from each organ and mostly from all three animals, infectious virus was recovered at this time point (Figure 3, lower right panel). To investigate the events that took place even earlier following viral inoculation, i.e., 3 dpi, and as hardly any virus was detectable in the day-5 animals, we increased the viral dose 40-fold for the animals to be sacrificed three days after virus application. In two of the three animals, viral DNA and infectious virus was found, one further animal remained negative for the two parameters in any organ. In the two animals being positive for virus, 6 × 10^8^–5 × 10^9^ viral DNA copy numbers, as well as considerable levels of infectious virus (10^5^ TCID_50_) were found in the nasal mucosa. In addition, one animal was positive for viral DNA in tonsil, tongue, lung and eyes, but loads were three or more orders of magnitude lower compared to the nasal mucosa (≤10^6^ copy numbers). Low numbers of infectious virus (~50 TCID_50_) were also found in tongue and esophagus (Figure 4).

### 3.7. Calpox Viral Load in Blood

Unexpectedly, calpox viral DNA was already detectable at a low level in blood (100 DNA copies/µL) of one of three animals (animal no. 3) inoculated with the high viral dose and sacrificed on day three after viral exposure. The same animal exhibited a higher frequency of calpox positive organs including those distally located from the inoculation site. Following inoculation with the lower viral dose, calpox viral DNA was detected in two of three animals at low levels at 7 dpi (animal no. 7 and 8), whereas the day-5 animals remained viral DNA negative in blood. Three days further in the infection course, only two out of three animals were still viremic, with a slightly increased viral load level in one animal. Highest calpox DNA levels were observed in the animals euthanized at 12 dpi ranging from 4.5 × 10^4^–8.4 × 10^5^ DNA copy numbers per microliter blood (Table 4).

## 4. Discussion

OPXV models are urgently needed to test new therapeutics and vaccines against emerging OPXV infections and to develop countermeasures against a potential bioterrorist smallpox attack. CPXV is suitable for studying human smallpox, because it shares 19 immunomodulatory genes with VARV, the agent of smallpox [43,49]. Furthermore, its infection route closely resembles that of natural smallpox transmission, in which through inhalation of infectious droplets the oral, nasal, or pharyngeal mucosae or, if deeper inhalation occurs, the lung are exposed and disease develops [50,51]. In previous studies, we demonstrated that in the calpox model, very low viral doses applied intranasally reliably led to lethal infection. Our model is highly suitable for studying OPXV-induced disease [45,48], as most of the clinical signs resemble those commonly found in smallpox. However, in those studies with calpox virus, we did not focus on the early phase of infection and the portal of virus entry which remain elusive so far. Until now, comprehensive analyses on pathological alterations were only carried out when the animals succumbed to disease. Also, nothing was known about the kinetics of calpox replication in organs. Therefore, we performed a study comprising serial necropsies during the asymptomatic phase of calpox infection in marmosets. At each defined time point, a broad spectrum of organs were collected and analyzed for histopathological alterations and the presence of virus to identify primary target cells and invaded organs. When the animals were inoculated with a low dose of calpox virus we observed only very mild histological alterations at day 5, 7 and 10. To increase the likelihood of finding first traces of virus and subtle morphological changes with our assays even earlier after viral exposure, a further three animals were inoculated with an approximately 40-fold higher dose and sacrificed at day three.

Our results demonstrate that the nasal epithelial cells serve as the principal target for primary replication after intranasal inoculation as we demonstrated at this site the presence of viral antigen within inclusion bodies by immunohistochemistry already at three dpi. The nasal mucosa is lined by a nasopharyngeal-associated lymphoid tissue (NALT), which contains many immune cells (B cells, T cells, macrophages, dendritic cells) [52]. Furthermore, the overlying epithelium is rich in microfold cells (M cells), which are responsible for uptake of viral antigen and transporting them to the deeper layers. Dendritic cells with antigen will migrate to the draining lymph node and present the antigen to T cells [53].

The presence of viral antigen within hyperplastic bronchial epithelium as early as three dpi suggests that a considerable amount of virus must have been inhaled after intranasal inoculation. However, keeping in mind that these animals received a high viral dose (3.5 × 10^5^ PFU), this could be a dose-related effect. Similar alterations were only found in one “low-dose” animal at day 7. Similarly, previous studies have shown that lung lesions could be seen in only 4 of 19 animals which were inoculated intranasally with different doses of calpox virus [48]. Diffuse pulmonary lesions (bronchopneumonia and interstitial pneumonia) with abundant viral antigens were detectable in our study from day 12 onwards, and seemed to be a consequence of systemic viral spread.

Destruction of respiratory epithelium results in an impaired clearance of microorganisms in the mucous blanket promoting secondary pulmonary infections. In our study, *Klebsiella pneumoniae* and hemolyzing *Escherichia coli* were found in lung tissue 12 and 16 dpi. Our results are in in line with data from mice infected intranasally with CPXV. In those animals, alveolar histiocytes and bronchiolar epithelial cells were observed to be the primary target cells [54,55]. In another study, infrequent bronchopneumonia was reported after intranasal and intradermal inoculation of cowpox virus into rats. However, secondary bacterial infections being responsible for pulmonal lesions could not be ruled out [56]. Severe pneumonia is known to be characteristic in OPXV infections [31,56,57,58,59,60] and especially in human smallpox, albeit it is not clear if it is directly virus-induced or due to secondary bacteremia [61]. However, our immunohistochemical results suggest that pulmonal lesions observed in our study are most probably virus-related. Contrary to aerosolized virus particles, which are more likely to be inhaled deeply and to reach distal parts of the lung, intranasally inoculated virus can cause tracheal and bronchial lesions related to the application of high doses [62], which we also found in our study.

It might be reasonable to assume that virus could cross the endothelial barrier of an adjacent artery leading to viremia and causing calpox-related lesions in more distant organs. Some tissues like buccal mucosa, spleen, axillary lymph node, adrenal gland, liver, lung, and skin showed in almost all animals that were immunoreactive for viral antigens from day 12 onwards, whereas other tissues like urinary bladder, kidney, tongue, and trachea were only occasionally affected.

Interestingly, viral antigen could be demonstrated only by day 7 in cortical regions of the submandibular lymph node, which is draining the inoculation site. Similar to this observation, infectious capripox virus could not be recovered from the draining lymph node until 8 dpi in a study with sheep and goats [63]. However, in our study, a mild hyperplastic response of the submandibular lymph node was observed already on day 3, suggesting immune activation associated with hyperplasia. This is further supported by the lack of severe histological lesions of other organs until day 12. After this time point, lymphoid necrosis replaced hyperplasia as part of collapse of the immune system allowing uncontrolled distribution of virus via macrophages to other tissues resulting in marked pathological alterations and high numbers of infected cells. Interestingly, lymphadenopathy is a classic alteration in many OPXV infections, but not in human smallpox [64,65].

Besides the submandibular lymph node, viral antigen could be detected in the spleen of one of the three day 7 animals via immunohistochemistry. Therefore, we assume these tissues represent sites of secondary replication. This hypothesis is supported by the detection of intracytoplasmic inclusion bodies in macrophages. In the submandibular lymph node, antigen was detected mainly in macrophages of the subcapsular sinus as well as in activated germinal centers. This is similar to observations made in a capripoxvirus study, although there were only few positive macrophages observed [64]. Infected macrophages might have reached the cortical regions via afferent lymph from the subcapsular sinus. It is likely that further infection of deeper regions of the lymph node at later time points happened through trafficking, via macrophages or dendritic cells [66].

Lesions in liver were absent to mild within the first 10 days and were restricted to multifocal to diffuse hepatocellular vacuolar degeneration without calpox specific immunohistochemistry. Observed vacuolar degeneration may be due to drugs used for anesthesia and euthanasia (ketamine, xylazine, pentobarbitone). Single calpox viral antigen positive Kupffer-cells by day 10 corroborate the importance of the mononuclear phagocyte system for pathogenesis. However, the livers of animals at a later stage of the disease showed dramatic alterations consisting of huge necrotic areas and focal hemorrhages. These findings are similar to those documenting MPXV-associated liver changes only by day 13. Contrary to our findings, Zaucha and co-workers only observed very little viral antigen within hepatocytes [66]. In another study with macaques receiving aerosolized MPXV, no virus-associated hepatic lesions were observed [35]. Embury-Hyatt et al. also noted the absence of hepatic (and splenic) lesions before day 11. This pattern is also typical for human smallpox, in which VARV does not cause drastic tissue destruction [64].

Animals which died spontaneously or were euthanized for ethical reasons by day 12, showed bacterial growth (hemolyzing *Escherichia coli* and *Klebsiella pneumonia*) in heart, lung, spleen, liver, and brain. This coinfection was probably due to immunosuppression, as it was always accompanied by lymphoid necrosis. Furthermore, poxvirus-infected macrophages which are originally attracted to fight the bacterial infection may in fact enhance dissemination of virus to areas that otherwise would be less affected [66]. Therefore, it can be assumed that acquired secondary bacteremia may lead to an accelerated disease course. This kind of secondary bacteremia was also found in several other OPXV models [49,56,66,67,68]. It is well known that CPXV manipulate the immune system, which in consequence could favor secondary bacterial infections [43,69,70,71]. Interestingly, secondary bacterial infections seen in studies with VARV, MPXV and CPXV were always associated with a hemorrhagic form of disease, which was also observed in animal models of human smallpox [49,61,66,67,68]. This was only partly true in our study as three of the four animals with bacterial growth in organs also showed signs of hemorrhagic disease. However, two monkeys suffering from hemorrhages in many organs were negative in bacterial culture. In our study, potential entry sites for bacteria were calpox-virus induced skin and mucosal lesions. Single animals (*n* = 3) showed mild lesions of the gastrointestinal tract, which could have also served as a portal for bacterial entry. Hemorrhage is a common finding in CPXV-induced lesions [31,62,72]. A study identified a viral protein that may inhibit the enzymes involved in blood coagulation resulting in hemorrhage [73]. The presence of viral antigen within endothelial cells could also result in damage of endothelial integrity and consequently hemorrhage. Furthermore, necrosis in liver could result in a reduced production of blood coagulation factors and thus in an increased hemorrhagic diathesis.

To determine the viral load in tissues two different methods were applied, i.e., measuring the infectious titers by an endpoint dilution assay and the viral DNA copy numbers by real-time PCR. Usually, the infectious titers were by several orders of magnitude lower than the viral copy numbers suggesting exceeding production of defective particles. Results from endpoint dilution assay and real-time PCR mostly matched with the pathological findings (e.g., nasal mucosa was negative for all 7 dpi animals, except in animal no. 8, which presented with highest viral load and mild histological lesions). The reasons for the discrepancies between pathological and virological findings (e.g., trachea was negative for all 12 dpi animals in immunohistochemistry and no histological lesions were found, but was virus positive, see Figure 4) most likely were attributable to the fact that for histology/immunohistochemistry different locations within one organ were analyzed compared to the material used for virological analyses. Thus, missing focal viral replication by either method was likely to happen. As expected, highest viral loads in tissue were observed in day-12 animals as they were close to or in one animal reaching mortality. Most efficient replication of calpox virus seemed to have taken place in nasal mucosa and the submandibular lymph node, but also in other lymph nodes and bone marrow. Less preferred targets for replication of calpox virus in the prefinal stage were the central nervous system, tongue, esophagus, and stomach, as indicated by low infectious titers and viral DNA copy numbers. These findings are consistent with other studies [39]. In two of the day-3 animals calpox DNA and/or infectious virus were detected in nasal mucosa, submandibular lymph node, tongue, esophagus, lung and eye, albeit calpox DNA copies were at least by three and infectious viral titers by five orders of magnitude higher in nasal mucosa compared to the viral load in the other organs. This strongly indicates that the nasal mucosa represents the portal of viral entry and primary replication site which is completely in line with the detection of calpox antigen in nasal epithelium by immunohistochemistry. Virus positivity in the other few organs was probably caused by swallowed or inhaled virus and attributable to the high viral load applied exceeding the inhaled variola dose sufficient to infect humans by 3500-fold [39]. Virus data from day-5 and day-7 animals support this evidence from the day-3 animals, as again considerably higher viral loads were observed in the nasal mucosa indicating preferential replication at this site independent of the inoculation dose. Based on the presence of calpox DNA copy numbers and infectious virus in a broad spectrum of organs from 7 dpi to 12 dpi we conclude that after local viral expansion at the inoculation site virus is systemically spread leading to viremia by day 7.

OPXV are known to be epitheliotropic and our results confirm this observation. We could detect calpox viral antigen in keratinocytes, urothelium, in scattered epithelial cells of the renal convoluted tubules, pneumocytes and in adrenal gland cells of the *zona fasciculata*. Interestingly, antigen was also detectable in endothelial cells mainly at the later stage of disease. Damage of endothelial cells can cause edema, hemorrhages and activates the extrinsic coagulation cascade leading to microthrombosis and disseminated intravascular coagulation [74].

Our results indicate that macrophages are likely to be involved in further spreading of virus. Recruitment of macrophages to virus-infected inflammation areas is a double-edged sword; it implies that macrophages can contribute to virus clearance, but they can also spread virus to further parts of the body [42]. The observed calpox virus loaded cells in the hamstring muscle of animal no. 21 could be explained by a calpox-unrelated lesion in the muscle, which could be due to intramuscular inoculation of ketamine for drawing blood samples. Macrophages which were attracted by the lesions might have been calpox virus infected causing a distribution of the virus to this untypical location. These macrophages carrying virus were also observed in many other OPXV studies [48,56,64,66,67,75]. An in vitro study with VACV confirms our observation, as monocytes were the most frequently infected cell population, followed by B cells, natural killer cells, and T cells [76].

The exact cause of death remains unknown, but seems to be related to multi-organ system failure, collapse of the immune system, and resulting opportunistic bacterial infections with sepsis. A “cytokine storm” is assumed to play also an important role [49]. However, due to the limited availability of reagents for common marmosets, the immunological details of calpox virus pathogenesis have been difficult to elucidate. This remains a key research priority for the future.

In agreement with the results of our previous studies [45], the incubation period was estimated as 11 to 12 days, which is comparable to human smallpox [77].

Mice models using Ectromelia Virus (ECTV) reveal similarities with our above-described animal model. As calpox virus, ECTV is infectious at low doses and causes high mortality [78]. The pathogenesis with localized replication followed by systemic spread is also comparable. ECTV is naturally transmitted via skin lesions [31]. In contrast, nothing is known about the natural transmission route of calpox virus. Bilateral conjunctivitis may be present at ECTV infections, which is not seen in our model [79]. However, the hepatotropic nature of ECTV is a further shared feature of the two models [31].

Intranasal infection of mice with VACV has also been extensively studied, and similar to our model, pulmonary lesions with peribronchial and perivascular inflammation and intra-alveolar edema are described [80]. Williamson et al. propose a pathogenesis which differs from our assumptions: According to him, respiratory infection is followed by viremia and infection of the central nervous system (CNS), although they cannot exclude direct CNS infection via cribriform plate [81].

**Figure 5:**
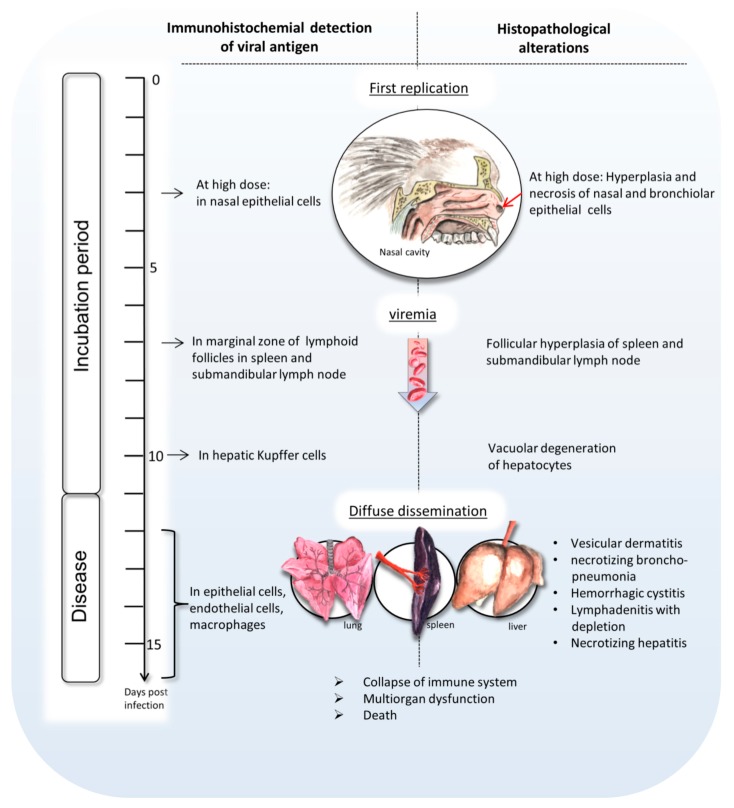
Model of calpoxviral pathogenesis.

In summary, this paper sheds light on the development of intranasal calpox virus infection in common marmosets during the incubation time and at the final disease stage (Figure 5). Our data indicate that early replication of calpox virus takes place in nasal epithelium starting at least at day 3 after intranasal inoculation, but maybe even earlier. Trafficking of infected macrophages presumably led to secondary replication in multiple organs including the submandibular lymph node and the spleen. Around day 7, Viremia, presumably monocyte associated, coincides with systemic dissemination of the virus proceeding in viral replication and typical calpox lesions in lung, liver, lymphatics, adrenal gland, mucocutaneous junctions, and skin. With the end of the incubation period of approximately 11 to 12 days, the animals start to develop a peracute progression of disease, and die within one to four days after the onset of clinical signs. The consistently fatal disease course indicates that our model is very suitable for the evaluation of new vaccines and therapeutics.

## Figures and Tables

**Table 1:** Age, gender, time, and mode of death in common marmosets *(Callithrix jacchus)* inoculated with calpox virus.

Animal No. ^a^	Age (Years)	Gender	Time of Death (dpi) ^b^
1	5	m	3
2	6	m	3
3	6	m	3
4	3	f	5
5	4	f	5
6	4	m	5
7	3	m	7
8	4	f	7
9	3	f	7
10	4	f	10
11	2	f	10
12	3	f	10
13	5	m	12
14	5	m	12
15	5	m	12
16	5	f	12
17	5	f	12
18	5	m	13
19	5	m	14
20	5	m	14
21	4	f	16

^a^ Animal no. 1–3 were infected intranasally with 3.5 × 10^5^ plaque forming units (PFU) of calpox virus, whereas animals no. 4–21 received 8.3 × 10^3^ PFU. ^b^ Animals no. 1–15 were euthanized at predefined time points (animal no. 15 met endpoint criteria); animals 16–21 were either euthanized for ethical reasons or have died spontaneously. f: female, m: male, dpi: days post inoculation.

**Table 2:** Main gross and histopathological findings during early, median and late phase of experimentally induced calpox virus infection of common marmosets (*Callithrix jacchus*).

dpi	3 ^a^	5	7	10	12	12–16
N	3	3	3	3	3	6
Gross lesion						
Splenic hyperplasia	2	2	1	3	3	2
Hyperplasia of submandibular lymph node	1	1	0	2	3	6
Cutaneous pox lesions	0	0	0	0	2	6
Hemorrhagic pneumonia	0	0	0	0	1	2
Mucosal petechiae on urinary bladder	0	0	0	0	1	3
Histological lesion						
Hyperplasia of nasal ciliated epithelium	2	1/2 ^b^	1/1 ^b^	1/2 ^b^	2/2 ^b^	3/3 ^b^
Lung Hyperplasia of bronchial epithelium	1	0	0	0	2	-
interstitial pneumonia	0	0	0	0	1	4
Spleen Follicular hyperplasia	1	2	0	3	1	-
Necrotizing splenitis with depletion	0	0	0	0	2	6
Submandibular lymph node Follicular hyperplasia	3	3	1/1 ^b^	3	-	1/4 ^b^
lymphadenitis with depletion	-	-	-	-	3	3/4 ^b^
Vesicular ulcerative dermatitis	0	0	0	0	2	6
Hemorrhagic/edematous cystitis	0	0	0	1	1	3/5 ^b^
Liver Hepatocellular degeneration	2	3	3	3	-	-
Necrotizing hepatitis	-	-	-	-	2	5

^a^ Animals in the first group received 3.5 × 10^5^ PFU, all other animals received 8.3 × 10^3^ PFU; ^b^ tissue could not be obtained from all animals; the number behind the slash indicates the total number of samples; Dpi: days post inoculation; N: number of animals.

**Table 3:** Histological lesions and detection of antigen by immunohistochemistry in selected organs of common marmosets inoculated with calpox virus.

dpi	Animal	Nasal Epithelium	Buccal Mucosa	Subm. LN	Trachea	Lung	Spleen	Liver	Urinary Bladder	Skin
		HE/IHC	HE/IHC	HE/IHC	HE/IHC	HE/IHC	HE/IHC	HE/IHC	HE/IHC	HE/IHC
3	1	0/−	0/−	1/−	0/−	1/+	0/−	1/−	0/−	2/−
3	2	1/(+)	0/−	1/−	1/+	1/+	0/−	0/−	0/−	0/−
3	3	2/++	0/−	1/−	0/−	1/+	1/−	1/−	0/−	0/−
5	4	0/−	0/−	1/−	0/−	0/−	1/−	1/−	0/−	0/−
5	5	na	1/−	1/−	0/−	1/−	1/−	1/−	1/−	0/−
5	6	1/(+)	0/−	1/−	0/−	1/−	0/−	1/−	0/−	0/−
7	7	na	0/−	na	0/−	0/−	0/−	1/−	0/−	0/−
7	8	1/+	0/−	1/+	0/−	0/−	0/+	1/−	1/−	0/−
7	9	na	0/−	na	0/−	1/+	0/−	1/−	0/−	0/−
10	10	1/−	0/−	1/−	1/−	1/+	1/−	2/−	2/−	0/−
10	11	na	1/−	1/+	2/−	1/−	1/+	1/+	0/−	0/−
10	12	0/−	0/−	1/++	0/−	1/−	1/−	1/−	0/−	0/−
12	13	1/−	0/−	2/(+)	0/−	1/+	1/+	2/+	1/−	3/+++
12	14	na	0/−	3/++	0/−	1/+	2/+	1/−	0/−	0/−
12	15	3/+++	2/++	2/++	0/−	2/++	2/+++	3/++	2/++	3/++
12	16	2/+	3/++	3/++	0/−	2/(+)	2/+	2/+++	3/+	3/++
12	17	na	1/+	2/++	0/−	1/+	3/+	2/++	0/−	3/++
13	18	1/+	1/+	na	2/++	1/++	3/+++	2/++	0/(+)	3/++
14	19	na	3/++	3/+++	0/−	0/(+)	3/+++	1/+	0/−	3/+++
14	20	2/++	0/−	na	0/−	2/++	1/(+)	2/−	na	3/+++
16	21	na	3/+++	2/+++	0/−	1/+	3/+++	2/+++	1/−	3/+++

na: not available, dpi: days post inoculation, HE: hematoxylin eosin staining, IHC: immunohistochemistry, subm.: submandibular; LN: lymph node, (+): single cells with immunohistochemical signal, +: <5% positive cells per high power field, ++: <50% positive cells per high power field, +++: 50–80% positive cells per high power field, 0: no histological lesion, 1: mild histological lesion, 2: moderate histological lesion, 3: severe histological lesion.

**Table 4:** Calpox viral DNA levels in blood of common marmosets serially sacrificed after intranasal calpox virus inoculation.

Animal #	Necropsy dpi *	Calpox DNA Copies/µL Blood on Day of Necropsy
1	3	neg
2	3	neg
3	3	115
4	5	neg
5	5	neg
6	5	neg
7	7	neg
8	7	14
9	7	55
10	10	neg
11	10	1.0 × 10^3^
12	10	83
13	12	3.6 × 10^5^
14	12	4.5 × 10^4^
15	12	8.4 × 10^5^

# Number; * dpi, day post inoculation.
